# Juvenile dermatomyositis and nephrotic syndrome: A case report and a mini literature review

**DOI:** 10.3389/fped.2023.1149785

**Published:** 2023-05-02

**Authors:** Weiran Zhou, Linlin Dong, Xuemei Liu, Chunhua Dong, Hongxia Zhang

**Affiliations:** Department of Pediatric Nephrology and Rheumatism and Immunology, Children's Hospital Affiliated to Shandong University, Jinan Children's Hospital, Jinan, China

**Keywords:** nephrotic syndrome, juvenile dermatomyositis, anti-NXP2, Adalimumab, case report

## Abstract

**Background:**

Renal involvement is rarely reported in juvenile dermatomyositis and may be caused by the toxic effects of myoglobinuria or an autoimmune reaction. We report a case of dermatomyositis and nephrotic syndrome in a child to explore the association between juvenile dermatomyositis and renal involvement.

**Case presentation:**

An 8-year-old girl with skin rash, edema, proximal muscle weakness predominantly involving the lower extremities, low-grade fever, and foamy urine was admitted to our hospital. Her laboratory tests met the criteria of nephrotic syndrome. She had elevated creatine kinase and lactate dehydrogenase and was diagnosed with juvenile dermatomyositis after electromyography and muscle MRI. Anti-NXP2 antibodies were positive. Her proteinuria was relieved soon after treatment with prednisone and methotrexate, but her muscle strength progressively decreased. The disease was relieved after pulse methylprednisolone treatment and mycophenolate mofetil, but recurred after drug reduction with mild proteinuria. Adalimumab was used for treatment and helped reduce the doses of glucocorticoid and mycophenolate mofetil.

**Conclusion:**

Juvenile dermatomyositis may be one of the rare causes of nephrotic syndrome. The mechanism involved in JDM combined with renal injury may be multifactorial. Autoantibodies may play important roles in both muscle and renal damage.

## Background

Juvenile dermatomyositis (JDM) is a rare idiopathic inflammatory disease with an incidence of 2–3/million/year (1). It is characterized by cutaneous findings and muscle weakness with various myositis-specific antibodies (MSAs) (2, 3). Anti-nuclear matrix protein 2 (anti-NXP2) antibodies, among the most important MSAs, have proven to be related to calcinosis, which is a high risk factor for poor treatment response and death (4, 5).

JDM patients without timely treatment could have physical disability, which seriously affects their quality of life and social participation (5). The treatment of refractory cases of idiopathic inflammatory myopathies is challenging. Biologic agents have been used for treatment or glucocorticoid (GC)-sparing effects (2, 6).

Multiple organs can be affected in JDM patients, especially the heart and lung (3). However, renal involvement in JDM is rarely reported. We report a case of recurrent anti-NXP2 JDM associated with nephrotic syndrome (NS).

## Case presentation

An 8-year-old girl was admitted primarily for skin rash and edema for one month. The patient was found to have proximal muscle weakness predominantly involving the lower extremities, low-grade fever, and foamy urine. There was no family history of the disease. The girl had a heliotrope rash, Gottron papules, and edema of the legs on physical examination. Manual muscle testing (MMT) scores (0–5) of her lower and upper limbs were 4 and 5 respectively.

**Figure 1:**
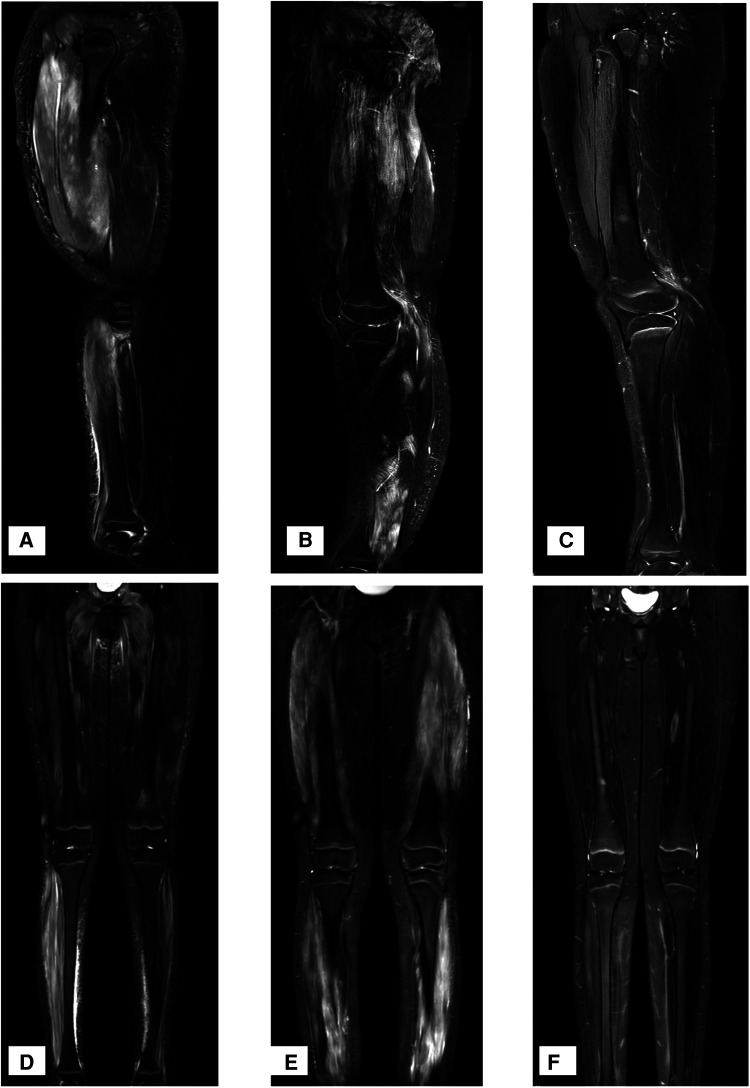
MRI T2W and stir-weighted images of the muscles at the time of onset. (**A,D**) T2W and Stir images at the time of onset, showing a high signal on T2-weighted and Stir images. (**B,E**) T2W and Stir images at the time of recurrence, showing extensive muscle damage. (**C,F**) T2W and Stir images one year after the application of Adalimumab.

Laboratory tests found that the girl had elevated creatine kinase (CK) and lactate dehydrogenase (LDH), massive proteinuria, decreased albumin and elevated cholesterol, as shown in Table 1. ANA, anti-DsDNA, anti-ENA, ANCA, anti-GBM, CMV-Ab, HCV-Ab, HBS-Ag, and HSV-DNA were negative. The urinary system and abdomen ultrasound and chest computed tomography scan did not show any abnormalities. Electromyogram showed narrowed motor unit potentials, polyphase potentials, increased irregular waves and recruitment potentials. Magnetic resonance imaging (MRI) showed high signals on T2-weighted and Stir images of the legs and no rhabdomyolysis or muscle necrosis, as shown in Figures 1A,D.

**Table 1:** Laboratory tests at the time of onset.

	Value	Normal range		Value	Normal range
CK (U/L)	1,665	21–220	Sodium (mmol/L)	132	135–145
CK-MB (U/L)	35	0–25	Potassium (mmol/L)	3.3	3.6–5.2
LDH (U/L)	630	114–240	Calcium (mmol/L)	1.77	2.2–3
SGOT (U/L)	107	0–38	Magnesium (mmol/L)	0.71	0.7–1
SGPT (U/L)	42	0–38	Serum Creatine (µmol/L)	41	25–74
WBC (×10^9^/L)	9.92	3.5–9.5	Nitrogen (mmol/L)	2.4	1.8–6.4
Hemoglobin (g/L)	149	120–140	Total protein (g/L)	42.8	60–80
Platelet (×10^9^/L)	321	125–350	Albumin (g/L)	21.2	35–50
CRP (mg/L)	3.13	0–10	Cholesterol (mmol/L)	6.78	3.15–5.0
ESR (mm/h)	29	0–20	Urine protein	4+	−
C3 (g/L)	1.22	0.5–1.8	UPCR	4.19	<0.2
C4 (g/L)	0.4	0.1–0.4	24 h urine protein (mg/day)	9,448.02	0–150

CK, creatine kinase; CK-MB, creatine kinase-MB; LDH, lactate dehydrogenase; SGOT, serum glutamic-oxaloacetic transaminase; SGPT, serum glutamic-pyruvic transaminase; WBC, white blood cell; CRP, C-reaction protein; ESR, erythrocyte sedimentation rate; C3, completement 3; C4, completement 4; UPCR, urine proteinuria-to-creatine ratio.

**Table 2:** Summary of the case reports of JDM with renal involvement.

References	Age	Sex	Clinical manifestation	Renal pathology	Immunofluorescence*	MSAs	Treatment	Renal outcome
Mantoo et al. (8)	10	Male	Hematuria and proteinuria	IgAN	IgA	–	GC, MTX	Improved
Civilibal et al. (9)	14	Female	Hematuria and proteinuria	IgAN	IgA	–	GC, MTX	Improved
Nickavar et al. (10)	7	Male	NS and renal dysfunction	FSGS with acute tubular injury	–	–		Improved
Soylu et al. (11)	10	Male	microscopic hematuria	MN	C1q	–	GC, MTX	Improved
Present case	8	Female	NS	–	–	NXP2	GC, MMF, Adalimumab	Improved

IgAN, IgA nephropathy; GC, glucocorticoid; MTX, methotrexate; NS, nephrotic syndrome; MN, membranous nephropathy; FSGS, focal segmental glomerular sclerosis; anti-NXP2, anti-nuclear matrix protein 2; MMF, mycophenolate mofetil.

* Immunofluorescence in kidney.

We made a diagnosis of JDM and NS. The MSA test showed that she had anti-NXP2 antibodies without other antibodies including Jo-1. The parents refused a renal biopsy.

Her urine protein decreased to 209.04 mg/24 h after 10 days of prednisone and methotrexate (MTX), but her muscle strength gradually decreased. The girl could not squat up, lie down, or get up, and she coughed after drinking. Her MMT scores (0–5) were 3 and 4 in her lower and upper limbs, respectively, and CK was 4,546 U/L. Methylprednisolone (MP) pulse therapy and intravenous immunoglobulin were given for 3 days, and her muscle strength did not significantly improve. MTX was replaced with mycophenolate mofetil (MMF) one month later, and her muscle strength gradually improved.

Twenty-one months later, the doses of MP and MMF were gradually reduced, and the skin rash and muscle weakness recurred. The 24 h urine protein was 227 mg (5.53 mg/m^2^/h, body weight 58 kg). MRI revealed extensive muscle damage and high signals in the muscles of the lower limbs, buttocks and pelvis in T2-weighted and Stir images (Figures 1B,E). The MP dosage was adjusted to 32 mg/day, and 40 mg adalimumab was subcutaneously injected every two weeks. The muscle strength of her limbs improved. MRI T2W and Stir-weighted images of the legs revealed decreased muscle damage one year after relapse (Figures 1C,F). Adalimumab was subcutaneously injected at 40 mg every two weeks for a total of 10 months and then every month for 5 months without side effects. The dosages of MMF and MP were decreased to 0.125 g and 2 mg per day, respectively.

## Discussion

The patient primarily presented with NS at the onset of the disease and was diagnosed with JDM with positive anti-NXP2 antibodies due to typical rash and extensive and progressive muscle damage. The proteinuria eased shortly after GC and MTX treatment. Adalimumab was used after relapse, helping to treat muscle damage and reduce the dose of GC.

As an inflammatory myopathy, typical clinical manifestations of JDM include the heliotrope sign, distal muscle weakness, increased muscle enzymes and muscle inflammation (3). Twenty percent of adult inflammatory myopathy patients have renal damage, which can manifest as IgA nephropathy, membranous nephropathy, focal segmental glomerular sclerosis, acute kidney injury, chronic kidney disease or even end-stage renal disease, and could occur before or after myopathy (7). However, reports of kidney damage in JDM patients have been limited to case reports. NS and renal function damage are rarely reported in cases of JDM.

We reviewed the literature and found four cases of JDM associated with renal involvement, as shown in Table 2. Clinical manifestations and renal outcomes varied. Mantoo et al. (8) and Civilibal et al. (9) separately reported a 10-year-old boy and a 14-year-old girl diagnosed with JDM and IgA nephropathy, respectively. Their haematuria and proteinuria decreased soon after treatment with GC and MTX for JDM. Nickavar et al. (10) reported a 7-year-old boy with NS and rapidly progressive renal dysfunction. Renal pathology suggested focal segmental glomerulosclerosis with acute tubular injury. The boy did not respond to MP pulse therapy, cyclophosphamide, or intravenous immunoglobulin. His renal function improved after plasma exchange, but NS continued. Subsequently, JDM symptoms developed. Soylu et al. described a 10-year-old boy with JDM and microscopic haematuria. Renal pathology showed membranous glomerulonephritis with C1q deposition (11). Renal outcome was not mentioned. In the present case, the girl had NS, which was quickly relieved after treatment with GC and MTX.

In adults, kidney damage is found at the same time as polymyositis or dermatomyositis in most cases, but there is also kidney damage months or even years before or after polymyositis or dermatomyositis (7). Cucchiari et al. reported that although most of the kidney damage has been improved after treatment, except for 4 patients who died, 7/27 polymyositis patients developed chronic kidney disease (CKD), including 4 patients with end-stage renal disease (ESRD). Three of the 19 dermatomyositis patients developed CKD (7).

The mechanism involved in JDM combined with renal injury may be multifactorial. Myoglobinuric rhabdomyolysis can cause acute tubular necrosis leading to acute kidney injury (12). In adults, acute kidney injury could occur in 16/150 inflammatory myopathy patients (13). T and B cells could be activated in JDM. A large number of inflammatory factors, especially interleukins and interferons are produced. These inflammatory factors can downregulate endothelial cell adhesion molecules, cause endothelial cell swelling, vascular obstruction and tissue ischaemia, and aggravate damage in muscles, kidneys and other organs (14, 15). Couvrat-Desvergnes et al. reported that 31 in 150 adults with inflammatory myopathy adults had CKD. Fourteen patients underwent a renal biopsy and all of the four ESRD patients had vascular lesions (13). In addition, autoantibodies, complements and cytokines that participate in the pathogenesis of JDM are also involved in many kinds of glomerulonephritis (16, 17). The deposition of immune complexes in the kidney can be found in JDM patients with chronic glomerulonephritis (8, 9, 11). Glomerulonephritis in JDM may be closely related to immune factors (10). Approximately 60%–95% of JDM patients have autoimmune antibodies which could suggest different clinical phenotypes and prognoses (3, 18, 19). Autoantibodies can directly act on vascular endothelial cells, leading to complement activation and vascular injury (20). The pathological study of muscle tissues of 23 children with JDM showed that muscle ischaemia was more severe in patients positive for anti-NXP2 antibodies, supporting the involvement of autoantibodies in vascular injury (21). The four JDM cases we reviewed did not identify the type of autoantibodies. However, myositis-specific antibodies and non-specific myositis-associated antibodies including SSA, SSB, RNP, Scl-70, pANCA were reported in most of adults with renal damage and inflammatory myopathy, of these antibodies, Jo-1 was the most common (13). In this case, the girl had massive proteinuria, progressive muscle damage and was positive for anti-NXP2 antibodies. Autoantibodies may play important roles in both muscle and renal damage.

Anti tumour necrosis factor (Anti-TNF) treatments such as adalimumab are now used in refractory JDM and have shown efficacy in the treatment of muscle and skin diseases. However, the evidence is limited to cohort studies and case reports (2, 6). TNF-α is overexpressed in children with JDM and is associated with prolonged JDM symptoms that require long-term immunosuppressive treatments (22). In addition, TNF-α-308 A alleles are associated with significantly increased serum interferon-α levels, and interferon-α is important for promoting inflammation in JDM patients (23). These findings suggest that anti-TNF therapy may be effective in treating JDM. A cohort study of sixty children with JDM in the UK showed that anti-TNF treatment, including infliximab and adalimumab, could improve skin and muscle disease. There was improvement in global disease activity for patients who received adalimumab. Twenty-five percent of patients had their treatments switched to anti-TNF treatment from infliximab to adalimumab to obtain a better improvement or due to adverse events and patient preference (24). Adalimumab was used for the currently reported patient when the disease relapsed after twenty-one months of treatment with GC and MMF. The symptoms were relieved after increasing the GC dose and adalimumab treatment. With the help of adalimumab, MP could be successfully reduced to 2 mg per day in one year without recurrence.

## Conclusion

JDM may be one of the rare causes of NS. The mechanism involved in JDM combined with renal injury may be multifactorial. Autoantibodies such as anti-NXP2 or Jo-1 may play important roles in both muscle and renal damage.

## Data Availability

The raw data supporting the conclusions of this article will be made available by the authors, without undue reservation.
